# Red Sea Bream Iridovirus Stability in Freeze–Thaw Cycles: Quantitative Assays of Infectious Particles

**DOI:** 10.3390/ani15121699

**Published:** 2025-06-09

**Authors:** Ji-Min Jeong, Gyoungsik Kang, Jae-Ok Kim, Jeong-Tae Lee, Chan-Il Park, Kyung-Ho Kim

**Affiliations:** 1 Aquatic Disease Control Division, National Fishery Products Quality Management Service (NFQS), Busan 49111, Republic of Korea; 2 Department of Aquatic Life Medicine, College of Marine Science, Gyeongsang National University, Tongyeong 53064, Republic of Korea; 3 Gyeongsangnam-Do Fisheries Safety Technology Institute, Tongyeong 53070, Republic of Korea; 4 Department of Marine Biology and Aquaculture, College of Marine Science, Gyeongsang National University, Tongyeong 53064, Republic of Korea

**Keywords:** aquatic virus, infectivity, Megalocytivirus, quantitative PCR assay, virus storage solutions, viability

## Abstract

Red sea bream iridovirus is a serious threat to farmed fish, but little is known about how repeated freezing and thawing affect its stability. This study investigated the effects of repeated freeze–thaw cycles on RSIV infectivity by comparing quantitative polymerase chain reaction (qPCR), viability qPCR (vqPCR), and 50% tissue culture infectious dose (TCID_50_) assays. While qPCR detected high amounts of viral DNA after multiple cycles, both viability qPCR and TCID_50_ revealed a significant loss of infectivity unless serum was present. Correlation analysis showed a high degree of agreement between vqPCR and TCID_50_, indicating their high compatibility for assessing viral infectivity. However, the correlations between qPCR and vqPCR, as well as between qPCR and TCID_50_, were significantly lower, suggesting that qPCR alone may overestimate viral infectivity by detecting non-infectious viral DNA. These results demonstrate the critical role of serum in preserving RSIV infectivity and highlight the superior accuracy of vqPCR and TCID_50_ in assessing viral infectivity compared with qPCR. This study emphasizes the importance of serum in storage media and suggests that combining vqPCR with TCID_50_ is a more reliable measure of RSIV infectivity than qPCR alone.

## Simple Summary

This study investigated the effects of repeated freeze–thaw cycles on red sea bream iridovirus. Repeated freeze–thaw cycles reduced the infectivity of red sea bream iridovirus unless serum was present. These findings highlight the importance of proper storage conditions for maintaining viral integrity and underscore the need for advanced methods to accurately assess infection risk in aquaculture.

## 1. Introduction

Red sea bream iridovirus (RSIV) is an enveloped double-stranded DNA virus (genus *Megalocytivirus*) that causes serious losses in aquaculture [1]. First identified in Japanese red sea bream (*Pagrus major*) in 1990 [2], it has since spread to over 30 marine fish species across East and Southeast Asia [3], leading to high mortality rates (60% to 100%) in susceptible species such as rock bream (*Oplegnathus fasciatus*), particularly when water temperatures are between 20 °C and 25 °C during the summer months [4,5]. Red sea bream iridoviral disease (RSIVD) is classified as a notifiable disease by the World Organisation for Animal Health (WOAH) due to its significant impact on aquaculture [1].

Viral samples are commonly preserved by freezing; however, while the stability of RSIV at ultra-low temperatures (−80 °C) has been well established, its viability after repeated freeze–thaw cycling remains uncertain [6]. Repeated freeze–thaw cycling can introduce physical and chemical stresses that can disrupt the viral envelope or capsid, leading to loss of infectivity [7,8,9,10,11,12]. Although PCR may still detect residual viral DNA after such cycles, this does not imply the presence of an infectious virus. The presence of non-infectious genomes can lead to an overestimation of viral infectivity, complicating the accurate determination of the true infectious viral load. [11,12]. While serum-supplemented storage solutions can protect viruses from freeze–thaw damage [13,14,15,16], few studies have evaluated the influence of different storage solutions on RSIV stability under repeated freeze–thaw events. Understanding these effects is essential for improving viral preservation and ensuring accurate viral load assessment.

Quantitative polymerase chain reaction (qPCR) is commonly used for RSIV detection [17,18]; however, it cannot distinguish between infectious virions and inactivated viral particles [19,20,21]. A positive qPCR result may indicate the presence of viral DNA from non-viable particles, potentially overestimating viral infectivity. To address this limitation, viability quantitative PCR (vqPCR) has been developed [22,23,24]. vqPCR uses a membrane-impermeant DNA-binding dye such as propidium monoazide (PMAxx) that binds to DNA from damaged viral particles and prevents amplification. This method correlates strongly with infectivity results from traditional cell culture assays, offering a more reliable tool for assessing viral viability and avoiding false positives from residual DNA.

This study aimed to evaluate the stability of RSIV under repeated freeze–thaw conditions and assess the influence of different storage solutions on viral infectivity. By comparing qPCR, vqPCR, and TCID_50_ assays, we aim to optimize viral preservation techniques and enhance the accuracy of RSIV diagnostic protocols, ultimately improving disease monitoring and control in aquaculture.

## 2. Materials and Methods

### 2.1. Cell Culture and Viral Production

*Pagrus major* fin cells [25] were cultured at 25 °C in Leibovitz’s L-15 medium (Gibco, Billings, MT, USA) supplemented with 10% fetal bovine serum (FBS; Gibco) and 1% antibiotic–antimycotic solution (A/A; Gibco). Confluent monolayers of *Pagrus major* fin cells were inoculated with RSIV genotype II (accession number: AY532608; RBIV-KOR-TY4), which was isolated from a diseased rock bream in South Korea [26], and maintained at 25 °C in L-15 medium supplemented with 2% FBS and 1% A/A. Infected cultures were harvested upon cytopathic effects, centrifuged (10,000× *g* for 10 min), filtered (0.45-µm), and stored at −80 °C.

### 2.2. Virus Titration

The viral titer was determined using a 50% tissue culture infectious dose (TCID_50_) assay. Ten-fold serial dilutions were prepared in L-15 medium supplemented with 2% FBS and 1% A/A, inoculated onto three well plates of a 96-well plate, and incubated at 25 °C. After 4 h, the inoculum was removed and replaced with a fresh medium. The Reed and Muench [27] method was used to calculate the TCID_50_.

### 2.3. Viral DNA Extraction and Quantitative PCR Assay

Viral DNA was isolated using the AccuPrep^®^ Genomic DNA Extraction Kit (Bioneer, Daejeon, Republic of Korea). RSIV was quantified using a previously described qPCR assay [28]. A cycle threshold (Ct) cut-off of 39.75 was applied for quantification using a real-time PCR platform [28].

### 2.4. Viability Quantitative PCR Assay

The vqPCR assay was performed as previously described [22]. Briefly, 200 µL aliquots from the RSIV storage solutions were treated with 75 µM PMAxx (Biotium, Hayward, CA, USA), incubated in the dark, and exposed to LED light for 30 min using a PMA-Lite™ LED Photolysis Device (Biotium). DNA was then extracted as in Section 2.3 and the nucleic acids were amplified using vqPCR to quantify viable viral particles. 

### 2.5. Preparation for Virus Stability Evaluation

The stability of RSIV at two viral concentrations (10^7.5^ and 10^5.5^ RSIV copies/mL) during repeated freeze–thaw cycles (0, 1, 3, 5, 7, 9, 12, 15, or 18) was assessed in three different storage solutions (L-15 medium with 10% FBS, L-15 medium, and PBS), using 50 mL conical tubes containing the viral solution. One cycle consisted of freezing at −80 °C for 24 h, thawing at 20 °C on a 100 rpm orbital shaker for 2 h, and refreezing for 24 h. Following each freeze–thaw cycle, samples were analyzed using qPCR, vqPCR, and TCID_50_ assays. All experiments were performed in triplicate for each test condition.

### 2.6. Statistical Analysis

RSIV decay was evaluated using a Bayesian regression model (Supplementary Material File S1) [29,30,31]. GraphPad Prism (version 10.4.1) was used for the statistical analyses. Pearson correlation coefficients were calculated to assess the relationships between qPCR, vqPCR, and TCID_50_ results. Agreement between the viral copy numbers or titers was assessed using the Bland–Altman analysis. Statistical significance is indicated by * *p* < 0.05, ** *p* < 0.01, *** *p* < 0.001, and **** *p* < 0.0001. All experimental measurements are reported as the mean of three replicates. 

## 3. Results

### 3.1. Effect of Freeze–Thaw Cycles on Viruses in Different Virus Storage Solutions

**Figure 1:**
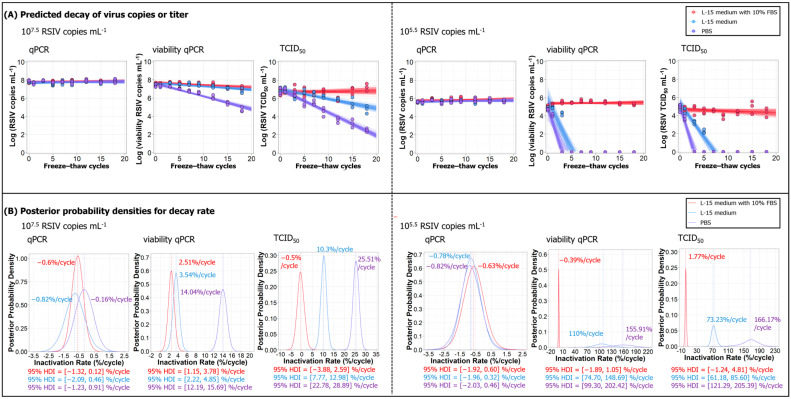
(**A**) Decay trend of RSIV over repeated freeze–thaw cycles for three different storage conditions: L-15 medium with 10% FBS (red), L-15 medium (blue), and PBS (purple). The viral stability was assessed using qPCR (left panel), vqPCR (middle panel), and TCID_50_ (right panel) at two viral concentrations: 10^7.5^ and 10^5.5^ RSIV copies/mL. For each condition, data from 18 freeze–thaw cycles were plotted, and Bayesian inference was applied to estimate the viral decay trend. The trend lines represent 100 random samples drawn from the posterior distribution of the Bayesian model, illustrating the possible decay trajectories with uncertainty across freeze–thaw cycles. Each point represents the log_10_ of viral copies or titer as estimated by the respective assay. (**B**) Posterior probability densities of the decay rate (inactivation rate) per cycle are presented for each condition, estimated from the Bayesian model. The 95% highest density interval (HDI) is shown for each posterior distribution, providing the range of plausible decay rates across cycles. The decay rate is expressed as the percentage decrease in viral titer per freeze–thaw cycle. The median decay rates and the 95% HDI for each condition are shown at the bottom of each plot, offering insights into the relative stability of RSIV under the different storage conditions.

We first examined the effect of repeated freeze–thaw cycles (up to 18 cycles) on RSIV stability in three different storage solutions: L-15 medium with 10% FBS, L-15 medium without FBS, and PBS. Two initial viral loads, high (10^7.5^ RSIV copies/mL) and low (10^5.5^ RSIV copies/mL), were tested, and the samples were analyzed using qPCR, vqPCR, and TCID_50_ assays (Figure 1A). The viral decay rate in freeze–thaw cycles was defined as the percentage decay per cycle (Figure 1B) (see Supplementary Material File S1).

In the qPCR analysis, the RSIV genome copy number remained stable throughout the experimental period, regardless of the storage solution or initial viral concentration (Figure 1A). The percentage decay rate per cycle as determined by qPCR did not decrease (Figure 1B). This suggests that the viral DNA remained intact even after repeated freeze–thaw cycles. In both vqPCR and TCID_50_ analyses, samples stored in L-15 medium with 10% FBS showed no significant decrease in viral load, even after 18 freeze–thaw cycles, regardless of the initial viral load. In contrast, identical samples stored in non-FBS L-15 medium or PBS exhibited a much more rapid decline in viral load with each cycle. High viral load samples remained detectable throughout all 18 cycles in L-15 medium and PBS, although the viral load gradually decreased after each cycle (Figure 1A). Specifically, vqPCR analysis indicated a viral decay rate per freeze–thaw cycle of 3.54% in L-15 medium and 14.04% in PBS, and, similarly, TCID_50_ analysis showed 10.3% and 25.51% declines per cycle in L-15 medium and PBS, respectively (Figure 1B). However, viral decay rates due to freeze–thaw cycles were especially pronounced in samples with low viral loads. The vqPCR analysis indicated that viruses stored in L-15 medium remained detectable up to the third freeze–thaw cycle (10^4.3±0.2^ RSIV copies/mL), whereas those stored in PBS were detectable only up to the first cycle (10^4±0.1^ RSIV copies/mL) (Figure 1A). After a single cycle, the viral decay rates reached 110% in L-15 medium and 155.91% in PBS (Figure 1B). Similarly, in the TCID_50_ analysis of low viral load samples, infectious virus in L-15 medium remained detectable up to the fifth freeze–thaw cycle (with titers near the detection limit; 10^2.2±0.2^ RSIV copies/mL) but was undetectable by the seventh. In PBS, infectivity was observed only in the first cycle (10^3.7±0.3^ RSIV copies/mL) and was undetectable by the third (Figure 1A). After a single freeze–thaw cycle, virus decay rates of 73.23% and 166.17% were observed in L-15 medium and PBS, respectively, indicating a significant reduction in viral titer (Figure 1B). Overall, these results suggest that the presence of serum protected RSIV from freeze–thaw-induced damage, whereas the absence of serum significantly compromised viral stability, especially at lower viral loads, where stability declines more rapidly.

### 3.2. Correlation Analysis Between Virus Stability Assays

**Figure 2:**
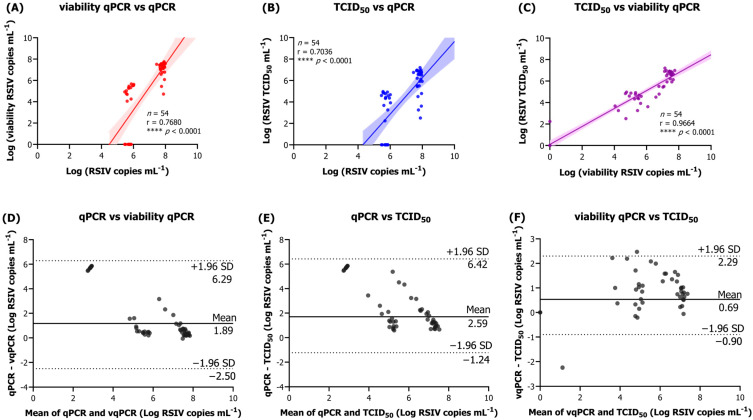
Correlation and agreement among qPCR, vqPCR, and TCID_50_ after repeated freeze–thaw cycles of RSIV. (**A**–**C**) Pearson correlation plots comparing log_10_ viral loads measured by qPCR, vqPCR, and TCID_50_ across all freeze–thaw conditions. Each point represents the mean of three technical replicates at a given cycle; shaded bands denote 95% confidence intervals for the regression lines. (**D**–**F**) The Bland–Altman plots show the agreement between the same assay pairs; solid lines indicate the mean difference and dashed lines indicate the ±1.96SD limits of agreement. Data combine high- and low-titer samples stored in L-15 medium ±10% FBS or PBS, illustrating the close concordance of vqPCR with TCID_50_ and the wider divergence of qPCR from both infectivity-based methods. **** *p* < 0.0001.

We performed repeated freeze–thaw cycles with high- and low-concentration viruses in three different storage solutions and analyzed the correlation between the three virus stability assays. The correlation between the three methods was significantly positive; however, the correlations between qPCR and TCID_50_ (Pearson’s r = 0.7036, **** *p* < 0.0001) and qPCR and vqPCR (r = 0.7680, **** *p* < 0.0001) were relatively low (Figure 2A,B). In contrast, the analysis of vqPCR and TCID_50_ (r = 0.9664, **** *p* < 0.0001) showed the strongest correlation (Figure 2C). The Bland–Altman analysis revealed that the mean differences among the three assays ranged from 0.69 to 2.59 (Figure 2D–F), with the smallest difference found between vqPCR and TCID_50_ (Figure 2F). These results indicate a high degree of agreement between the vqPCR and TCID_50_ assays.

## 4. Discussion

Despite multiple freeze–thaw cycles, qPCR continued to detect high viral genome copy numbers, indicating that viral DNA remained intact. In contrast, vqPCR and TCID_50_ assays showed significant declines in infectivity, highlighting that while DNA may remain detectable, infectivity is compromised.

Viral samples stored in PBS and non-FBS L-15 medium exhibited pronounced loss of infectivity, particularly at low viral loads. In contrast, L-15 medium containing 10% FBS retained significantly higher infectivity across repeated cycles, supporting the role of serum in protecting viral particles from ice crystal damage [16,17]. Bayesian regression modeling quantified these differences, showing that the estimated mean infectivity loss per freeze–thaw cycle was as low as approximately 2.51% in L-15 medium containing 10% FBS, compared to a maximum of approximately 110% in L-15 medium alone and 166.17% in PBS, based on vqPCR and TCID_50_ analyses. Fetal bovine serum, rich in growth factors, hormones, amino acids, proteins, vitamins, inorganic salts, and antibodies, is commonly used to enhance viral stability during low-temperature storage and freeze–thaw cycles [32,33]. It has been reported that while ice crystals formed during freezing can physically damage the viral structure, the serum components surround the virus, preventing direct contact between the ice crystals and the viral surface, thereby protecting the virus from loss of infectivity [34,35,36]. These results are consistent with previous studies on other viruses, such as the respiratory syncytial virus [16], oncolytic measles virus [37], and equine influenza virus [38], all of which showed enhanced stability when stored in FBS-containing media. The addition of serum significantly stabilizing the virus is consistent with previous reports [39,40]. Therefore, RSIV samples should be stored in serum-containing media to minimize titer loss, preserve infectivity, and ensure more reliable viral stability for downstream analyses.

Relying solely on qPCR may overestimate the quantity of infectious viruses [41,42,43,44,45]. In contrast, vqPCR amplifies the viral DNA only if the envelope or capsid of the viral particle remains intact. If the virus is damaged, dyes, such as PMAxx, bind to nucleic acids and inhibit DNA amplification. The TCID_50_ assay is useful for assessing viral infectivity but can be limited by the sensitivity of the cell line used [46]. In contrast, vqPCR is efficient for the rapid detection and quantification of infectious viruses [22]. Our results showed a strong correlation between vqPCR and TCID_50_ (Pearson’s r = 0.9664, *p* < 0.0001) in most samples, indicating that vqPCR more effectively reflects the infectivity status of the virus, which qPCR alone cannot fully assess. As such, these methods are complementary. However, in samples with low viral concentrations stored in L-15 medium, vqPCR detected the virus up to the third freeze–thaw cycle, whereas TCID_50_ detected it up to the fifth. This discrepancy is likely due to envelope-damaged but still infectious particles being excluded by the dye. The results support past findings that vqPCR is more efficient and accurate than cell culture methods for assessing RSIV viability in seawater [22]. In marine environments, physical and chemical stresses can cause rapid degradation of the external structure of viruses [47]. In this context, vqPCR-based assessment of viral integrity in environmental seawater could provide advantages over cell culture methods that require virus concentration and dilution [22,24].

## 5. Conclusions

In conclusion, freeze–thaw cycles minimally affect RSIV DNA detection by qPCR but markedly compromise viral infectivity. Serum-supplemented storage media significantly protects RSIV from freeze–thaw-induced damage. Moreover, combining vqPCR with TCID_50_ offers a reliable measure of true infectivity, thus reducing overestimation of viral risk. These findings underscore the importance of proper sample storage and advanced detection methods for the accurate assessment of RSIV infectivity, ultimately enabling more effective disease monitoring and control strategies in aquaculture. 

## Data Availability

The original contributions presented in this study are included in the article/Supplementary Material. Further inquiries can be directed to the corresponding authors.

## Supplementary Materials

The following supporting information can be downloaded at https://www.mdpi.com/article/10.3390/ani15121699/s1, File S1. RSIV Decay under Different Freeze–Thaw Conditions: A Bayesian Regression Model.
