# Supplementary material for: Red Sea Bream Iridovirus Stability in Freeze–Thaw Cycles: Quantitative Assays of Infectious Particles
Source: Animals (Basel). 2025 Jun 9;15(12):1699. doi: 10.3390/ani15121699 (PMC12189574; doi:10.3390/ani15121699)
Supplement: Supplementary file 1 [file animals-15-01699-s001.zip › animals-3652474-supplementary.pdf]

Red Sea Bream Iridovirus Stability in Freeze–Thaw Cycles: Quantitative Assays of Infectious Particles

Supplementary Material

Bayesian regression model for viral decay

A Bayesian linear regression model was applied to estimate the effect of repeated freeze-thaw cycles on the infectious titer of red sea bream iridovirus (RSIV) [1]. The response variable was the $\log_{10}$-transformed viral titer, denoted y_{ij}, modeled as a linear function of the number of freeze-thaw cycles (x_{ij}). This approach quantifies the average decline in the $\log_{10}$ titer per additional freeze-thaw cycle while accounting for experimental variability and censoring due to the detection limit of the assay.

Notation and cut-off value

Let y_{ij} represent the $\log_{10}$ titer for sample i after j freeze-thaw cycles, and let $x_{ij} = j$ be the corresponding number of cycles. The experimental detection limit was 0.5 $\log_{10}$, meaning any titer measurements ≤ 0.5 were below the reliable quantification range. We treated such values as left-censored at 0.5 $\log_{10}$ (i.e., the true value is known only to be ≤0.5). This censoring approach allowed for the inclusion of samples with undetectable virus in the Bayesian analysis without underestimating the true decline.

Linear model specification

We assumed a simple linear relationship between freeze-thaw exposure and virus titer. For each sample i and cycle count j, the model is

$$y_{ij} = \beta_0 + \beta_1 \cdot x_{ij} + \varepsilon_{ij}$$

where β_0 is the intercept (mean $\log_{10}$ titer with zero freeze-thaw cycles), β_1 is the regression slope (change in $\log_{10}$ titer per cycle), and ε_{ij} ~ Normal (0, σ) represents the residual error

term. A negative value of β_1 indicates the decay of viral titer with each additional freeze-thaw cycle, as expected. In this formulation, each observed titer contributes information about β_0, β_1, and the overall variability σ. Left censoring was handled by the model's likelihood: for any observation where y_{ij} was censored at 0.5, the likelihood accounted for all possible true values below 0.5, rather than a single-point estimate.

Prior distributions and model fitting

We chose weakly informative prior distributions for all model parameters, reflecting reasonable assumptions regarding their scales without strongly constraining them. Specifically, we placed a Normal (5.5, 4) prior on β_0 (centered around a plausible initial titer of 5.5 $\log_{10}$ with broad uncertainty), a Normal (0, 2) prior on β_1 (allowing for a wide range of possible slopes centered at zero change), and a Cauchy (0, 2) prior on σ (a heavy-tailed prior restricting the residual standard deviation to a realistic range). These priors are intentionally broad to provide slight regularization but let the data drive the posterior estimates.

The model was fitted using the brms package in R 4.4.2, which uses the Stan's Hamiltonian Monte Carlo sampler. We ran four parallel Markov chain Monte Carlo (MCMC) chains, each for 2000 iterations with the first 1000 iterations used for the warm-up (burn-in). This yielded 4 × 1000 = 4000 posterior draws for the inference. Convergence of the MCMC was confirmed by inspecting trace plots and ensuring all $\hat{R}$ values were close to 1.00 (all $\hat{R} < 1.01$) and effective sample sizes were high. Posterior parameter estimates are reported as the median and 95% highest-density interval (HDI) of the posterior distribution for each parameter, which provides a clear summary of the credible range of each estimate. Inference and summarization were facilitated by the Tidybayes package in R, which was used to extract the posterior medians and HDIs.

Viral decay rate and posterior

To interpret the effect size on a percentage scale, we derived the percentage decay per cycle (D) from the slope parameter. We defined $D = -100 \times \beta_1$, which represents the percentage

decrease in viral titer with each additional freeze-thaw cycle. For example, if β_1 = -0.10 (a drop of 0.10 $\log_{10}$ per cycle), this corresponds to approximately a 20% reduction in actual titer per cycle. We computed the posterior distribution of D by transforming each posterior sample of β_1, and we summarized D using its median and 95% HDI to convey the most probable decay rate and the uncertainty around it.

The adequacy of the model was evaluated using posterior predictive simulations. Specifically, we generated simulated titer data from the fitted model and compared them with the observed data across all cycle numbers. Posterior predictive checks indicated that the model could reproduce the key features of the empirical data, including the mean trend and frequency of censored (undetectable) observations at higher cycle counts. This suggests that the linear model with censoring provided a reasonable description of the freeze-thaw decay dynamics.

References

Gelman, A.; Carlin, J.B.; Stern, H.S.; Dunson, D.B.; Vehtari, A.; Rubin, D.B. *Bayesian Data Analysis*, 3rd ed.; CRC Press: Boca Raton, FL, USA, 2013. https://doi.org/10.1201/b16018.
